# The Association between the Platelet to White Blood Cell Ratio and Chronic Kidney Disease in an Aging Population: A Four-Year Follow-Up Study

**DOI:** 10.3390/jcm12227073

**Published:** 2023-11-13

**Authors:** Yang Xiong, Qian Zhong, Yangchang Zhang, Feng Qin, Jiuhong Yuan

**Affiliations:** 1 Department of Urology and Andrology Laboratory, West China Hospital, Sichuan University, Chengdu 610041, China; cqmu_xy@163.com; 2 Department of Endocrinology, West China Hospital, Sichuan University, Chengdu 610041, China; 3 Department of Public Health, Capital Medical University, Beijing 100054, China

**Keywords:** aging, chronic kidney disease, CHARLS, platelet to white blood cell ratio

## Abstract

Introduction: The platelet to white blood cell ratio (PWR) has been reported to be a prognostic factor for some diseases, such as subarachnoid hemorrhage. However, the association between the PWR and chronic kidney disease (CKD) remains unknown. To investigate the cross-sectional and longitudinal association between the PWR and CKD, this study was performed. Methods: This study used datasets from a national prospective cohort in China (China Health and Retirement Longitudinal Study). A retrospective cohort from 2011 to 2015 was constructed. The PWR was stratified as a categorical variable according to tertiles (T1–T3 groups). CKD was defined as an estimated glomerular filtration rate < 60 mL min^−1^/1.73/m^2^. Univariate and multivariate logistic regressions and restricted cubic spline regression were adopted to assess the linear and non-linear association between the PWR and CKD. Propensity score matching was used to balance the discrepancies between covariates. Subgroup and interactive analyses were performed to explore potential interactive effects of covariates. Missing values were interpolated using random forest. The PWR was also stratified according to the median and quartiles as sensitivity analyses. Results: A total of 8600 participants were included in this study. In the full model, the odds ratios (ORs) of prevalent CKD were 0.78 (95% CI = 0.62–0.97, *p* < 0.05) for the T2 group and 0.59 (95% CI = 0.46–0.76, *p* < 0.001) for the T3 group. There were significant interactive effects of marital status and smoking in the PWR–CKD association (both *p* for interaction < 0.05). An L-shaped, non-linear association was detected between the PWR and prevalent CKD in the overall population, participants ≥ 60 years, and females subgroups (all *p* for non-linear < 0.05). All sensitivity analyses supported the negative association between the PWR and prevalent CKD. In the 2011–2015 follow-up cohort, the ORs of incident CKD were 0.73 (95% CI = 0.49–1.08, *p* > 0.05) and 0.31 (95% CI = 0.18–0.51, *p* < 0.001) for the T2 and T3 groups, respectively, in the full model. Conclusions: A high PWR is associated with a reduced risk of prevalent and incident CKD. The PWR may serve as a predictor for CKD, facilitating the early identification and intervention of kidney function decline.

## 1. Introduction

Chronic kidney disease (CKD) is defined as a condition characterized by kidney structural abnormalities or sustained impairment in kidney function for a period of three months or more [1]. This condition can manifest as pathological kidney damage, abnormalities in blood or urine components, or abnormal findings in imaging studies. Additionally, CKD can also be diagnosed when the glomerular filtration rate (GFR) of the kidneys is less than 60 mL/min/1.73 m^2^ for a duration of three months or longer and there is no clear underlying cause for this decline in kidney function [2]. CKD is typically classified into stages based on the severity of kidney impairment, with higher stages indicating a more advanced disease [3]. CKD has become a global public health challenge, marked by an increasing incidence, substantial economic burdens, and a heightened morbidity and mortality rate [4]. According to the World Health Organization’s reports, CKD has surged to become the 10th leading cause of death worldwide, with a staggering 1.3 million reported deaths by 2019, representing a significant rise over the past two decades [5]. In addition to traditional risk factors such as hypertension, diabetes, and old age [6], chronic inflammation is also considered a significant factor that cannot be ignored in the progression of kidney diseases [7]. Inflammation plays a crucial role in kidney injury and the loss of nephrons. It does so by amplifying inflammation or oxidative stress, generating pro-inflammatory cytokines, and altering the immune system [8,9]. The study by Munoz Mendoza and colleagues showed that elevated levels of interleukin-6 (IL-6) and the C-reactive protein (CRP) are independent risk factors for mortality in CKD [10]. However, measuring inflammatory markers such as interleukins can be challenging in primary medical care settings. Therefore, it is important to search for simpler inflammatory markers to predict the risk of CKD [11].

In recent years, novel inflammation markers derived from peripheral blood cell analysis, such as the monocyte-to-lymphocyte ratio (MLR), neutrophil-to-lymphocyte ratio (NLR), platelet-to-lymphocyte ratio (PLR), and systemic immune-inflammation index (SII), have gained significant attention due to their convenience, repeatability, and cost-effectiveness [5,12]. They have become prognostic indicators for various chronic diseases, such as cardiovascular diseases and tumors, reflecting the degree of systemic low-intensity inflammation [11]. Recently, the relationship between these novel inflammation markers, MLR, NLR, and PLR, and the progression of CKD has been studied. Muresan et al. [5] found that NLR, MLR, and PLR, determined upon admission, had strong predictive capabilities for 30-day, all-cause mortality in ESKD patients requiring RRT for at least six months. In a prospective analysis of 938 patients diagnosed with CKD stages I–IV by Yuan et al. [13], it was discovered that patients with an NLR ≥ 2.09 had a statistically significant increase in the progression of ESKD. A follow-up study based on a small cohort suggested that a higher MLR was a powerful independent predictor of all-cause and cardiovascular mortality in hemodialysis patients [14]. Additionally, a study from Peru suggested a relationship between high NLR, PLR, and all-cause mortality in CKD patients. Patients with elevated NLR or PLR showed twice the risk of mortality compared to those with normal ratios [15].

The platelet to white blood cell ratio (PWR), calculated as the absolute platelet count divided by the absolute white blood cell (WBC) count measured in peripheral blood, is a recently discovered hematologic inflammation marker. It has been reported to be used in predicting short-term postoperative outcomes in patients undergoing renal malignancy surgery [16]. Furthermore, it has been demonstrated to serve as an independent predictor of clinical outcomes in acute promyelocytic leukemia [17], ischemic stroke [18], and pyogenic liver abscesses [19]. Apart from their hemostatic function, platelets can trigger and exacerbate inflammation by interacting with immune cells and secreting pro-inflammatory cytokines. Additionally, several research findings suggest that PWR can reflect the severity of systemic inflammation [20]. Despite being considered a critical biomarker in various diseases, the correlation between PWR and CKD has not yet been established. Therefore, the purpose of this study is to address this question using data from the China Health and Retirement Longitudinal Study (CHARLS).

## 2. Materials and Methods

### 2.1. Data Sources and Included Populations

In this study, surveys from CHARLS were analyzed. As a longitudinal survey, CHARLS is designed to investigate economic and health statuses, accessibility to medical service, biomarkers, healthcare and insurance, etc., in Chinese residents aged ≥ 45 years. It was initiated in 2011 and the participants were followed up every 2–3 years. To date, four national waves in 2011, 2013, 2015, and 2018 have been performed. CHARLS adopted multistage stratified probabilities proportional to the size sampling method to obtain a representative aging population. A detailed description of CHARLS can be found within the official publication [21] or website (http://charls.pku.edu.cn/, accessed on 11 October 2023). This study was approved by the ethical review board of Peking University (IRB 00001052-11014). Written and oral informed consent was obtained from all participants before participating in this project.

**Figure 1:**
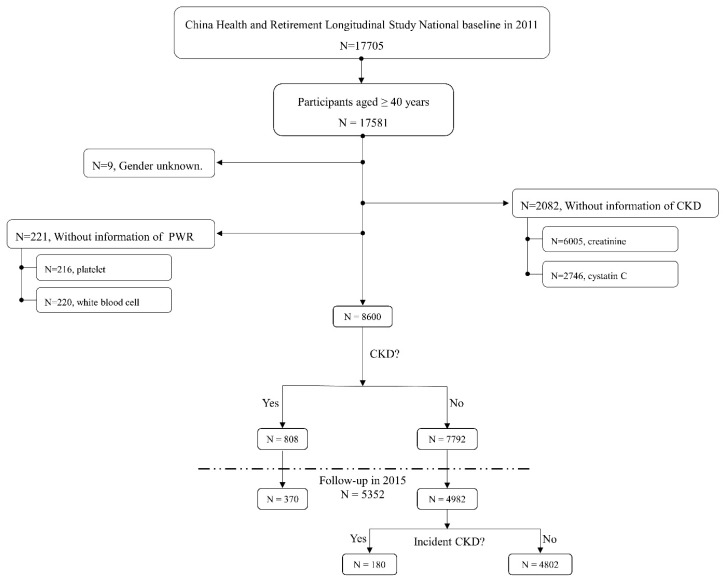
Study design and analysis strategy. In CHARLS, we first investigated the cross-sectional association between CircS and prevalent CKD in 2011 and the longitudinal association was explored in a four-year follow-up survey from 2011 to 2015. PWR: platelet/white blood cell ratio; CKD: chronic kidney disease.

Only the 2011 and 2015 waves explored the concentration of creatinine and cystatin C necessary to diagnose CKD. Thus, in this study, the 2011 baseline survey was used to investigate the cross-sectional association between the PWR and prevalent CKD. The included participants were followed up for four years until 2015 to explore the longitudinal association between the PWR and incident CKD. As summarized in Figure 1, participants aged < 40 years with unknown gender and inadequate information to calculate the PWR and diagnose CKD were excluded. Finally, a total of 8600 participants were included after data cleansing.

### 2.2. Measurements of the PWR and CKD

The PWR is calculated as the amount of platelet (×10^9^/L)/white blood cells (×10^9^/L). To define CKD, we first calculated the estimated glomerular filtration rate (eGFR) based on the 2012 CKD-EPI creatinine–cystatin-C equation [22]. Patients with an eGFR < 60 mL min^−1^/1.73/m^2^ were diagnosed as having CKD [21].

### 2.3. Collection of Venous Blood and Measurements of Blood Biomarkers

To collect qualified venous blood, the participants were asked to fast overnight. Well-trained medical staffs were responsible for blood collection in collaboration with local nurses. Three tubes of venous blood were collected from each respondent using a standard protocol [23]. The first 2 mL tube was used for complete blood count analysis on automated analyzers. The second 6 mL tube was used to collect whole blood, which was then centrifuged to obtain plasma. The plasma was temporarily stored at −20 °C. This tube was used to determine the concentration of high-sensitivity C-reactive protein, lipid panel (total, high-density lipoprotein, low-density lipoprotein, and triglyceride), blood glucose, creatinine, uric acid, and cystatin C. The third 2 mL tube of whole blood was collected for the HbA1C assay. The second and third tubes were finally shipped to the Capital Medical University via a cold-chain shipping company for final determination. Blood creatinine was assessed using the picric acid method and cystatin C was assessed using an immunoturbidimetric assay. The detailed process of blood biomarker determination can be found in a previous study [23].

### 2.4. Evaluation of Covariates

Demographic variables, lifestyle variables, medical history, and blood biomarkers were used as covariates in this study. Demographic variables included age (years), educational levels (literate and illiterate), gender (male and female), marital status (married/cohabitating and others,) and body mass index (BMI). The illiterate group referred to participants with lower educational levels than elementary school. The others group in marital status referred to the divorced/separated/widowed. The BMI was stratified into four groups: <18.5, 18.5–24.0, 24.0–28.0, and ≥28.0 kg/m^2^. Lifestyle factors consisted of sleep duration (0–6, 6–8, and >8 h), afternoon nap (yes or no), cigarette consumption (current, never, or ex-smoker), and alcohol consumption (more than once a month, less than once a month, and never). Medical history was composed of depression, hypertension, and hyperuricemia. Depression was assessed using the Center for Epidemiological Studies Depression Scale-10 (CESD-10) questionnaire [24]. Participants with scores ≥ 10 were diagnosed with depression. Hypertension was defined as systolic pressure ≥140 mmHg or diastolic pressure ≥90 mmHg or drug treatment of hypertension. Hyperuricemia was defined as blood uric acid >420 μmol/L for males and >360 μmol/L for females. Blood biomarkers included low-density lipoprotein (mg/dL), high-density lipoprotein (mg/dL), total cholesterol (mg/dL), triglycerides (mg/dL), blood glucose (mg/dL) and high-sensitivity C-reactive protein (mg/L) as one previous study did [25]. Diabetes was defined as fasting glucose ≥126 mg/dL (7.0 mmol/L), Hba1C ≥ 6.5%, random plasma glucose ≥200 mg/dL (11.1 mmol/L), self-reported history, and/or the use of anti-diabetic medications [25].

### 2.5. Statistical Analyses

The PWR was stratified according to tertiles (T1, T2 and T3 groups). The data were presented as mean ± standard error (SD) for continuous measures with a normal distribution and median (25–75% quantiles) with a non-normal distribution and n (%) for categorical measures. The differences across tertiles were tested using a *t*-test, Wilcoxon test, or Chi-square test according to the data types. To evaluate the cross-sectional association between the PWR and CKD, binary logistic regression was used. A set of models were constructed by adjusting different covariates: Model 1—crude model; Model 2—adjusting for demographic characteristics including age, gender, marital status, educational levels, and BMI; Model 3—further adjusting for lifestyle factors including cigarette and alcohol consumption, sleep duration, and afternoon nap; Model 4—adjusting for medical histories including depression, hypertension, and hyperuricemia; Model 5—adjusting for blood biomarkers including low-density lipoprotein, high-density lipoprotein, total cholesterol, triglycerides, blood glucose, and high-sensitivity C-reactive protein. The participants were followed up to 2015 to investigate the longitudinal association between the PWR and CKD.

To verify the robustness of our findings, some sensitivity analyses were performed. First, the PWR was used as a continuous variable to confirm the cross-sectional and longitudinal associations. In addition, restricted cubic spline (RCS) regression with three knots was employed to explore the dose–response associations between the PWR and CKD. For non-linear association, the threshold value was determined and the PWR was divided as a binary variable according to the threshold value. The propensity score matching (PSM) method was used to balance the covariates between the <threshold value group and ≥threshold value group. The matching method was nearest-neighbor matching with a caliper of 0.03. Second, we performed subgroup and interactive analyses to investigate the potential interactive effects of covariates. Third, considering that there is no consensus in the equation for estimating eGFR, we also used the 2009 CKD-EPI creatinine equation to calculate the eGFR as a sensitivity analysis [22]. Fourth, given that there were 0–15% missing values in different covariates (Figure S1), we interpolated the dataset using multivariate imputation by chained equations based on random forest methods [24]. The interpolated dataset was analyzed to verify the findings. Finally, given the lack of rationality in stratifying PWR according to tertiles, we also stratified the PWR according to the median and quartiles via sensitivity analyses.

In this study, all data were analyzed using R 4.0.2 software (R Foundation for Statistical Computing, Vienna, Austria). A *p*-value < 0.05 (two-sided) indicates statistical significance.

## 3. Results

### 3.1. Characteristics of Participants in the 2011 Baseline Survey

After data cleansing, 8600 participants were included in the 2011 baseline survey (Figure 1). The baseline characteristics are summarized in Table 1. Participants with a higher PWR tended to be younger, female, and literate; have less cigarette and alcohol consumption; display higher low-density lipoprotein, total cholesterol, and eGFR; and show lower blood glucose, rates of diabetes, triglycerides, uric acid, and C-reactive protein (all *p* < 0.05).

### 3.2. The Cross-Sectional Association between the PWR and Prevalent CKD

Participants with a higher PWR had a lower risk of prevalent CKD (Table 2). As a continuous variable, the PWR was associated with a decreased risk of prevalent CKD in all five regression models. The ORs ranged from 0.976 to 0.983 (all *p* < 0.001) for every one-unit increase in the PWR. As a categorical variable, the OR of CKD for the T2 group was 0.70 (95% CI = 0.59–0.82, *p* < 0.001) and that for the T3 group was 0.49 (95% CI = 0.41–0.59, *p* < 0.001) in the crude model. In the full model (Model 5), the T2 group had a 0.78-fold (95% CI = 0.62–0.97, *p* < 0.05) risk of CKD and the T3 group had a 0.59-fold (95% CI = 0.46–0.76, *p* < 0.001) risk of CKD. All five models supported the decreased risk of CKD for a high PWR (all *p* for trend < 0.001).

### 3.3. Association between the PWR and Prevalent CKD in Subgroup and Interactive Analyses

**Figure 2:**
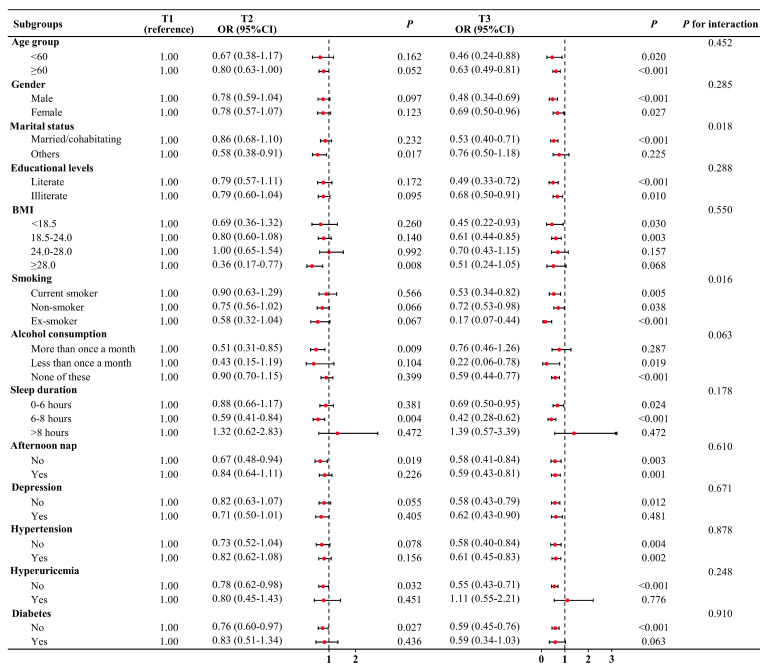
Association between the PWR and prevalent CKD in subgroup and interactive analyses. The T1 group was used as the reference group. In the multivariable logistic regression models, covariates were adjusted as in Model 5 in previous analyses except for subgroup variables.

A significant decreased risk of CKD was observed in most of the subgroups (Figure 2). However, the ORs for the T3 group were 0.76 (95% CI = 0.50–1.18, *p* = 0.225) for the widowed/separated/divorced group, 0.70 (95% CI = 0.43–1.15, *p* = 0.157) for participants with a BMI of 24–28 kg/m^2^, 0.76 (95% CI = 0.46–1.26, *p* = 0.287) for participants drinking more than once a month, 1.39 (95% CI = 0.57–3.39, *p* = 0.472) for participants sleeping > 8 h, 1.11 (95% CI = 0.55–2.21, *p* = 0.776) for participants with hyperuricemia, and 0.59 (95% CI = 0.34–1.03, *p* = 0.063) for patients with diabetes. The insignificance may be attributed to the relatively limited sample size in these subgroups (Table 1). Notably, there were significant interactive effects of marital status and smoking in the PWR–CKD association (both *p* for interaction < 0.05).

### 3.4. The Dose–Response Association between PWR and Prevalent CKD

**Figure 3:**
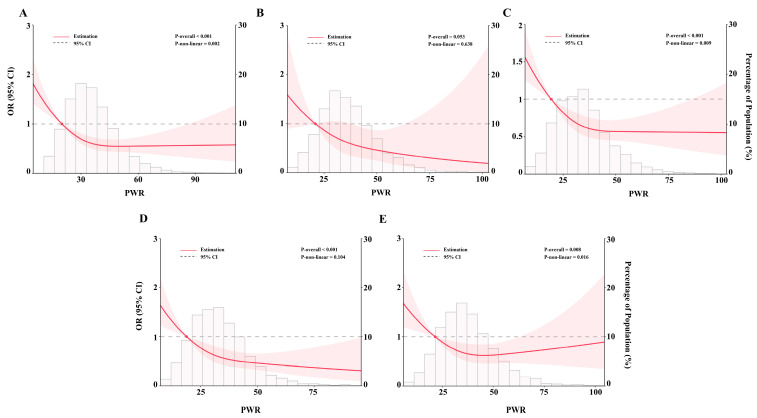
The dose–response association between the PWR and prevalent CKD. Restricted cubic spline regression was used to evaluate the dose–response relationship between the PWR and prevalent CKD. In the overall population (**A**), covariates were adjusted as in Model 5. In participants aged < 60 years (**B**) and ≥60 years (**C**), age was not adjusted. In males (**D**) and females (**E**), gender was not adjusted. The red line shows the odds ratio and the pink area shows the 95% confidence interval.

To investigate the dose–response association between the PWR and prevalent CKD, a RCS regression was performed. In Figure 3A, an L-shaped, non-linear association was detected between the PWR and prevalent CKD in the overall population (*p* for non-linear = 0.002). The inflection point was 30.89. We further recoded the PWR as a binary variable according to the inflection point and performed regression analysis (Table 3). After adjusting for different covariates, the ORs for prevalent CKD were 0.60 (95% CI = 0.52–0.69, *p* < 0.001), 0.67 (95% CI = 0.56–0.80, *p* < 0.001), 0.67 (95% CI = 0.56–0.81, *p* < 0.001), 0.67 (95% CI = 0.55–0.81, *p* < 0.001), and 0.67 (95% CI = 0.55–0.82, *p* < 0.001) in the crude model, Model 2, Model 3, Model 4, and the full model (Model 5), respectively. After PSM, univariable logistic regression also found a 0.83-fold (95% CI = 0.69–0.99, *p* = 0.040) risk of CKD for participants with a PWR ≥ 30.89.

We further explored the dose–response association in different age groups (<60 years and ≥60 years) and gender (males and females). The L-shaped, non-linear association was not observed in participants aged < 60 years (*p* for non-linear = 0.638, Figure 3B) but was detected in participants aged ≥ 60 years (*p* for non-linear = 0.009, Figure 3C). In Figure 3D, a linearly downward association was detected in males (*p* for overall < 0.001), which was non-linear in females (*p* for non-linear < 0.05, Figure 3E).

### 3.5. Sensitivity Analyses to Verify the Association between the PWR and Prevalent CKD

First, as shown in Figure S1, there were 0% to 15% missing values in different covariates. Therefore, the missing values were interpolated using the random forest method and reanalyzed, identifying similar findings as in Table 2. In Table S1, as a continuous variable, the ORs ranged from 0.976 to 0.982 (all *p* < 0.001) for every one-unit increase in PWR. As a categorical variable, all five models supported the decreased risk of prevalent CKD for a high PWR (all *p* for trend < 0.001).

Second, given that there is no consensus in the equation for estimating eGFR, we also used the 2009 CKD-EPI creatinine equation to calculate the eGFR using sensitivity analysis. In Table 4, as a continuous variable, the ORs ranged from 0.979 to 0.989 (all *p* < 0.05) for every one-unit increase in PWR. As a categorical variable, the ORs for the T3 group were 0.52 (95% CI = 0.40–0.67, *p* < 0.001), 0.65 (95% CI = 0.48–0.87, *p* < 0.01), 0.64 (95% CI = 0.47–0.86, *p* < 0.01), 0.67 (95% CI = 0.48–0.93, *p* < 0.05), and 0.67 (95% CI = 0.48–0.94, *p* < 0.05) in the crude model, Model 2, Model 3, Model 4, and the full model (Model 5), respectively. Similarly, all five models supported the decreased risk of CKD for a high PWR (all *p* for trend < 0.05). The negative correlation between the PWR and CKD was still valid using the new definition.

Third, we also stratified the PWR according to the median and quartiles as sensitivity analyses (Table S2). Stratified according to the median, a higher PWR was significantly associated with a lower risk of prevalent CKD. The ORs of the PWR > median group ranged from 0.61 to 0.69 in all five regression models (all *p* < 0.001). Stratified according to quartiles, a decreased risk for prevalent CKD was detected in all groups (Q2, Q3, and Q4 groups) and all five regression models (all *p* < 0.01 and *p* for trend < 0.001).

### 3.6. The Longitudinal Association between the PWR and Incident CKD

The included participants were followed up for four years until 2015 to explore the longitudinal association between the PWR and incident CKD. In Table 5, it was found that participants with a higher PWR had a lower risk of incident CKD. As a continuous variable, the PWR was associated with a decreased risk of incident CKD in all five regression models. The ORs ranged from 0.977 to 0.982 (all *p* < 0.001) for every one-unit increase in PWR. As a categorical variable, the ORs for the T3 group were 0.41 (95% CI = 0.28–0.60, *p* < 0.001), 0.39 (95% CI = 0.25–0.61, *p* < 0.001), 0.35 (95% CI = 0.22–0.56, *p* < 0.001), 0.30 (95% CI = 0.18–0.51, *p* < 0.001), and 0.31 (95% CI = 0.18–0.51, *p* < 0.001) in the crude model, Model 2, Model 3, Model 4 and the full model (Model 5), respectively. All five models supported the decreased risk of incident CKD for a high PWR (all *p* for trend < 0.001).

## 4. Discussion

In comparison to the traditional clinical risk factors associated with CKD, the PWR has garnered relatively less attention in the realm of public health. However, this population-based prospective analysis demonstrated that a high PWR was significantly associated with a reduced risk of prevalent and incident CKD in middle-aged and older persons in China, as revealed in both cross-sectional and longitudinal examinations. To the best of our knowledge, this study represents the first instance of the PWR being identified as a predictive factor for prevalent and incident CKD, thereby adding valuable insights to the body of knowledge concerning modifiable risk factors for kidney health.

In the baseline survey, the participants with a higher PWR were generally younger, more likely to be female, literate, non-smokers, non-drinkers, and had favorable lipid profiles and lower levels of blood glucose, triglycerides, uric acid, and C-reactive protein. These characteristics may indicate a healthier lifestyle and fewer risk factors for CKD, which could be related to their reduced CKD risk. In particular, we observed an L-shaped, non-linear relationship of the PWR with prevalent CKD in the overall population and participants aged ≥60 years. This means that as the PWR increased, the risk of prevalent CKD decreased; however, beyond a certain threshold (the inflection point, which was determined to be 30.89 in this study), further increases in the PWR did not lead to additional reductions in CKD risk. It is important to identify this threshold to optimize the potential benefits of the PWR in CKD prevention. We also found a linearly downward association in males, which was non-linear in females. The reasons behind these gender-specific differences could be multifactorial and may relate to hormonal, genetic, or lifestyle factors that influence the interaction between the PWR and CKD risk differently in men and women.

Similar to our research findings, many studies have also indicated that higher PWR values may serve as a protective factor against the development and progression of various diseases, such as pancreatic cancer [26], cirrhosis [27], and liver failure [28]. A retrospective cohort study comprising 269 untreated pancreatic cancer patients discovered that a declined PWR was independently linked to unfavorable outcomes in individuals with pancreatic cancer [26]. Similarly, in another retrospective study that recruited 131 patients with HBV-associated decompensated cirrhosis (HBV-DeCi), a lower PWR was correlated with an elevated risk of mortality, and the PWR emerged as an independent predictor of mortality in HBV-DeCi patients [27]. Wang et al. performed a retrospective analysis of data from 800 patients diagnosed with aneurysmal subarachnoid hemorrhage upon admission. Their study revealed that those patients with a PWR < 15.69 upon admission had an increased probability of developing postoperative pneumonia [20]. Zhao et al. [29] retrospectively analyzed clinical data from 338 patients with cytogenetically normal acute myeloid leukemia at the time of disease diagnosis and found that the PWR was an independent prognostic predictor in acute myeloid leukemia. Beyond these studies, our study shows that PWR is a predictive factor for prevalent and incident CKD, which is beneficial for the early identification and intervention of kidney function decline.

The mechanistic relationship between the PWR and CKD remains to be elucidated. It is well known that inflammation plays a crucial role in the development and progression of CKD. Uncontrolled inflammation can result in damage to glomerular, tubular, and interstitial structures, leading to renal hemodynamic imbalances and an inability to regulate blood pressure [30]. The PWR might serve as an alternative indicator of patients’ baseline health status. From our study, it appears that patients with a lower PWR are more likely to have additional risk factors such as advanced age, smoking, alcohol consumption, and hyperlipidemia. These physiological and biochemical abnormalities could potentially lead to inappropriate activation of inflammatory pathways. Platelets are circulating anucleated cells that play a crucial role in hemostasis [31]. In patients with CKD, in addition to a decrease in platelet count, several abnormalities in platelet function have been observed [32]. A reduction in platelet adhesion and aggregation in nondialyzed CKD patients was reported over 40 years ago [33,34]. Recently, a systematic review showed platelet dysfunction and reduced platelet aggregation in CKD, along with a prolonged bleeding time [35]. This suggests the possibility of platelet exhaustion in CKD, a concept where elevated platelet activation under pathophysiological conditions leads to a secondary loss of platelet function [36]. This concept may contribute to the explanation of why CKD patients are at an increased risk of both thrombosis and bleeding. Therefore, a high PWR may be associated with better platelet function. Normal platelet function is essential for maintaining vascular health and coagulation. A higher PWR may indicate more effective platelet aggregation and coagulation responses, thereby reducing the risk of bleeding. This could contribute to lowering the risk of CKD, as bleeding or coagulation issues may have a negative impact on kidney health [32].

Moreover, there is a growing body of literature highlighting the role of circulating platelets in modulating various pathophysiological processes beyond thrombosis, such as inflammatory processes and immune responses [37]. However, the precise pathophysiological roles of platelets in generating inflammatory and hemostatic complications in CKD patients remain largely unexplored. Over the last six decades, studies on platelet function in CKD patients have produced conflicting reports, with some indicating reduced activation, some indicating hyperactivation, and others indicating unchanged platelet activation [38]. A growing body of literature emphasizes the role of platelets in modulating the immune response during inflammation [37]. Platelets can influence leukocyte function directly through cell–platelet adhesion and indirectly by releasing soluble mediators and microparticles [39]. The most unifying concept that ties these events together is that platelets may participate in modulating systemic inflammation in the CKD state [38]. Platelet release can be modulated by inflammatory cytokines as a mechanism for rapid recovery [15]. Patients with CKD often exhibit a condition characterized by chronic, low-grade inflammation [40]. This persistent proinflammatory environment has been linked to increased platelet reactivity and, in some cases, a diminished response to antiplatelet therapy. Hence, chronic, low-level inflammation could potentially impact platelet function in CKD [32].

Additionally, an elevated WBC count is a widely recognized predictor of CKD progression [41]. Elevated WBC counts often indicate inflammation, while low lymphocyte counts may reflect immunosuppression. In patients with CKD, the combination of a high WBC count and a low lymphocyte count is associated with a poorer prognosis [42,43,44]. Elevated WBC levels lead to the production of various pro-inflammatory cytokines, a condition referred to as hypercytokinemia. This can result in damage to vascular endothelial cells and contribute to the promotion of renal sclerosis and fibrosis [45]. However, it is essential to emphasize that the PWR is not a stable marker of the inflammatory state and depends on when it is measured. The values of the PWR can vary if the patient is in a state of acute inflammation that affects these values.

There are several important limitations that need to be noted. First, the follow-up duration may not have been long enough, which could potentially limit the incidence of CKD to some extent. Similar studies with longer follow-up periods are necessary. Second, we used eGFR to define the incidence of CKD instead of directly measuring GFR. However, eGFR is widely accepted due to the impracticality and cost associated with direct GFR measurement. Third, this study did not assess white blood cell subtypes such as neutrophils and lymphocytes. Fourth, as an important confounding factor, albuminuria was not measured and included as a covariate in our analyses, which should be addressed in future studies. Fifth, CKD is defined based on abnormal kidney function for a period of three months or more. In our study, given the large sample size in a national cohort, eGFR was not repeatedly measured after three months, which may bring bias to our results. Additionally, since all participants in this study were Chinese, the findings may not be applicable to other regions and ethnicities. Finally, this is an observational study, which means that further investigations are needed to establish causality.

In summary, a high PWR reduces the risk of CKD in middle-aged and older individuals. For CKD patients, it is important to monitor their hematological parameters, especially platelet and WBC counts. If they also have chronic low-grade inflammation, treatment targeting inflammation should be considered. Abstinence from alcohol and cigarettes and the absence of hyperuricemia are potential protective factors but require further confirmation in longitudinal studies.

## 5. Conclusions

In conclusion, our study provides evidence that a high PWR is associated with a decreased risk of CKD. This suggests that the PWR could potentially serve as a practical and cost-effective tool for early identification and intervention in CKD patients. Future research should delve into the underlying mechanisms of the PWR–CKD relationship and further validate its clinical utility.

## Figures and Tables

**Table 1:** Characteristics of participants in 2011 baseline survey.

Characteristics	Tertiles of PWR			Total N = 8600	*p*
	T1 N = 2867	T2 N = 2867	T3 N = 2866		
Age (years)	61.45 ± 10.20	60.38 ± 10.16	59.42 ± 10.16	60.42 ± 10.21	<0.001
Gender					<0.001
Male	1595 (55.63%)	1359 (47.40%)	1095 (38.21%)	4049 (47.08%)	
Female	1272 (44.37%)	1508 (52.60%)	1771 (61.79%)	4551 (52.92%)	
Marital status					0.345
Married/cohabitating	2311 (80.61%)	2354 (82.11%)	2332 (81.37%)	6997 (81.36%)	
Others	556 (19.39%)	513 (17.89%)	534 (18.63%)	1603 (18.64%)	
Educational levels					0.017
Literate	1407 (49.08%)	1467 (51.17%)	1514 (52.83%)	4388 (51.02%)	
Illiterate	1460 (50.92%)	1400 (48.83%)	1352 (47.17%)	4212 (48.98%)	
BMI (kg/m^2^)					0.912
<18.5	174 (7.21%)	178 (7.33%)	190 (7.87%)	542 (7.47%)	
18.5–24.0	1269 (52.61%)	1278 (52.64%)	1254 (51.95%)	3801 (52.40%)	
24.0–28.0	695 (28.81%)	676 (27.84%)	688 (28.50%)	2059 (28.38%)	
≥28.0	274 (11.36%)	296 (12.19%)	282 (11.68%)	852 (11.75%)	
Cigarette consumption					<0.001
Current smoker	1055 (36.89%)	881 (30.85%)	719 (25.20%)	2655 (30.98%)	
Non-smoker	1510 (52.80%)	1705 (59.70%)	1940 (68%)	5155 (60.16%)	
Ex-smoker	295 (10.31%)	270 (9.45%)	194 (6.80%)	759 (8.86%)	
Alcohol consumption					<0.001
Drink more than once a month	768 (26.86%)	747 (26.16%)	633 (22.19%)	2148 (25.07%)	
Drink less than once a month	226 (7.90%)	219 (7.67%)	187 (6.56%)	632 (7.38%)	
None of these	1865 (65.23%)	1890 (66.18%)	2032 (71.25%)	5787 (67.55%)	
Sleep duration (hours)					0.577
0–6	1401 (51.72%)	1405 (51.60%)	1355 (50.00%)	4161 (51.11%)	
6–8	1070 (39.50%)	1096 (40.25%)	1113 (41.07%)	3279 (40.27%)	
>8	238 (8.79%)	222 (8.15%)	242 (8.93%)	702 (8.62%)	
Afternoon nap					0.936
No	1246 (45.62%)	1256 (45.81%)	1240 (45.32%)	3742 (45.58%)	
Yes	1485 (54.38%)	1486 (54.19%)	1496 (54.68%)	4467 (54.42%)	
Depression					0.159
No	1696 (66.22%)	1725 (66.86%)	1662 (64.42%)	5083 (65.83%)	
Yes	865 (33.78%)	855 (33.14%)	918 (35.58%)	2638 (34.17%)	
Hypertension					0.065
No	1420 (56.22%)	1441 (56.98%)	1507 (59.33%)	4368 (57.51%)	
Yes	1106 (43.78%)	1088 (43.02%)	1033 (40.67%)	3227 (42.49%)	
Hyperuricemia					<0.001
No	2654 (92.57%)	2684 (93.62%)	2747 (95.85%)	8085 (94.01%)	
Yes	213 (7.43%)	183 (6.38%)	119 (4.15%)	515 (5.99%)	
LDL (mg/dL)	113.04 ± 34.88	118.31 ± 34.59	118.06 ± 35.15	116.47 ± 34.95	<0.001
Total Cholesterol (mg/dL)	191.26 ± 38.31	195.33 ± 38.67	193.26 ± 39.07	193.28 ± 38.72	<0.001
HDL (mg/dL)	50.71 ± 15.58	51.20 ± 15.31	51.28 ± 15.02	51.06 ± 15.31	0.305
Blood glucose (mg/dL)	112.55 ± 41.03	111.14 ± 36.30	107.88 ± 31.64	110.52 ± 36.57	<0.001
Diabetes					0.001
No	2378 (82.94%)	2390 (83.36%)	2469 (86.21%)	7237 (84.17%)	
Yes	489 (17.06%)	477 (16.64%)	395 (13.79%)	1361 (15.83%)	
C-reactive protein (mg/L)	1.23 (0.63–2.71)	1.07 (0.56–2.29)	0.89 (0.50–1.79)	1.06 (0.56–2.26)	<0.001
Triglycerides (mg/dL)	137.14 ± 104.02	133.77 ± 108.29	127.07 ± 94.03	132.66 ± 102.36	<0.001
eGFR (mL min^−1^/1.73 m^2^)	81.20 ± 18.50	84.48 ± 17.85	86.79 ± 17.17	84.16 ± 17.60	<0.001
CKD					<0.001
No	2508 (87.48%)	2607 (90.93%)	2677 (93.41%)	7792 (90.60%)	
Yes	359 (12.52%)	260 (9.07%)	189 (6.59%)	808 (9.40%)	
Platelets (×10^9^/L)	157.05 ± 51.51	212.28 ± 52.95	264.24 ± 77.90	211.18 ± 75.87	<0.001
White blood cell (×10^9^/L)	7.21 ± 2.16	6.24 ± 1.54	5.24 ± 1.26	6.23 ± 1.87	<0.001
PWR index	22.15 ± 5.03	34.10 ± 3.02	51.26 ± 12.63	35.84 ± 14.40	<0.001

**Table 2:** The cross-sectional association between PWR and prevalent CKD.

Models	PWR (Continous)	PWR (As Tertiles)			
	OR (95% CI)	T1 (Reference)	T2 Group OR (95% CI)	T3 Group OR (95% CI)	*p* for Trend
Model 1	0.976 (0.971–0.982) ***	1.00	0.70 (0.59–0.82) ***	0.49 (0.41–0.59) ***	<0.001
Model 2	0.982 (0.975–0.988) ***	1.00	0.80 (0.65–0.98) *	0.56 (0.45–0.71) ***	<0.001
Model 3	0.981 (0.974–0.988) ***	1.00	0.81 (0.66–0.99) *	0.56 (0.45–0.71) ***	<0.001
Model 4	0.983 (0.975–0.991) ***	1.00	0.77 (0.62–0.96) *	0.59 (0.46–0.75) ***	<0.001
Model 5	0.983 (0.976–0.991) ***	1.00	0.78 (0.62–0.97) *	0.59 (0.46–0.76) ***	<0.001

* *p* < 0.05; *** *p* < 0.001. Model 1—crude model; Model 2—adjusting for demographic characteristics including age, gender, marital status, educational levels, and BMI; Model 3—further adjusting for lifestyle factors including cigarette and alcohol consumption, sleep duration, and afternoon nap; Model 4—adjusting for medical histories including depression, hypertension, and hyperuricemia; Model 5—adjusting for blood biomarkers including low-density lipoprotein, high-density lipoprotein, total cholesterol, triglycerides, diabetes, and high-sensitivity C-reactive protein.

**Table 3:** The cross-sectional association between the PWR and prevalent CKD (as binary according to RCS regression).

Models	PWR < 30.89	PWR ≥ 30.89	*p*
	Reference	OR (95% CI)	
Model 1	1.00	0.60 (0.52–0.69)	<0.001
Model 2	1.00	0.67 (0.56–0.80)	<0.001
Model 3	1.00	0.67 (0.56–0.81)	<0.001
Model 4	1.00	0.67 (0.55–0.81)	<0.001
Model 5	1.00	0.67 (0.55–0.82)	<0.001
PSM	1.00	0.83 (0.69–0.99)	0.040

The inflection point was determined according to RCS regression. Model 1—crude model; Model 2—adjusting for demographic characteristics including age, gender, marital status, educational levels, and BMI; Model 3—further adjusting for lifestyle factors including cigarette and alcohol consumption, sleep duration, and afternoon nap; Model 4—adjusting for medical histories including depression, hypertension, and hyperuricemia; Model 5—adjusting for blood biomarkers including low-density lipoprotein, high-density lipoprotein, total cholesterol, triglycerides, diabetes, and high-sensitivity C-reactive protein. PSM: propensity scores matching.

**Table 4:** The cross-sectional association between the PWR and CKD using 2009 CKD-EPI creatine equation.

Models	PWR (Continous)	PWR (As Tertiles)			
	OR (95% CI)	T1 (Reference)	T2 Group OR (95% CI)	T3 Group OR (95% CI)	*p* for Trend
Model 1	0.979 (0.971–0.987) ***	1.00	0.77 (0.61–0.97) *	0.52 (0.40–0.67) ***	<0.001
Model 2	0.986 (0.977–0.995) **	1.00	0.87 (0.66–1.13)	0.65 (0.48–0.87) **	0.005
Model 3	0.985 (0.976–0.995) **	1.00	0.87 (0.67–1.15)	0.64 (0.47–0.86) **	0.004
Model 4	0.988 (0.978–0.998) *	1.00	0.83 (0.61–1.11)	0.67 (0.48–0.93) *	0.016
Model 5	0.989 (0.979–0.999) *	1.00	0.85 (0.63–1.15)	0.67 (0.48–0.94) *	0.021

* *p* < 0.05; ** *p* < 0.01; and *** *p* < 0.001. Model 1—crude model; Model 2—adjusting for demographic characteristics including age, gender, marital status, educational levels, and BMI; Model 3—further adjusting for lifestyle factors including cigarette and alcohol consumption, sleep duration, and afternoon nap; Model 4—adjusting for medical histories including depression, hypertension, and hyperuricemia; Model 5—adjusting for blood biomarkers including low-density lipoprotein, high-density lipoprotein, total cholesterol, triglycerides, diabetes, and high-sensitivity C-reactive protein.

**Table 5:** The longitudinal association between the PWR and incident CKD.

Models	PWR (Continous)	PWR (As Tertiles)			
	OR (95% CI)	T1 (Reference)	T2 Group OR (95% CI)	T3 Group OR (95% CI)	*p* for Trend
Model 1	0.977 (0.965–0.988) ***	1.00	0.67 (0.48–0.95) *	0.41 (0.28–0.60) ***	<0.001
Model 2	0.977 (0.964–0.991) ***	1.00	0.69 (0.48–1.01)	0.39 (0.25–0.61) ***	<0.001
Model 3	0.973 (0.960–0.987) ***	1.00	0.70 (0.48–1.03)	0.35 (0.22–0.56) ***	<0.001
Model 4	0.972 (0.957–0.987) ***	1.00	0.73 (0.49–1.08)	0.30 (0.18–0.51) ***	<0.001
Model 5	0.972 (0.958–0.987) ***	1.00	0.73 (0.49–1.08)	0.31 (0.18–0.51) ***	<0.001

* *p* < 0.05; *** *p* < 0.001. Model 1—crude model; Model 2—adjusting for demographic characteristics including age, gender, marital status, educational levels, and BMI; Model 3—further adjusting for lifestyle factors including cigarette and alcohol consumption, sleep duration, and afternoon nap; Model 4—adjusting for medical histories including depression, hypertension, and hyperuricemia; Model 5—adjusting for blood biomarkers including low-density lipoprotein, high-density lipoprotein, total cholesterol, triglycerides, blood glucose, and high-sensitivity C-reactive protein.

## Data Availability

The datasets used and/or analyzed during the current study are available from the corresponding author upon reasonable request.

## Supplementary Materials

The following supporting information can be downloaded at: https://www.mdpi.com/article/10.3390/jcm12227073/s1, Table S1: The cross-sectional association between PWR and prevalent CKD after interpolation. Table S2: The cross-sectional association between PWR and prevalent CKD (as binary or quartiles). Figure S1: The missing values of covariates. (A) The percentages of missing values of covariates; (B) The combinations of missing values of covariates.
