# Supplementary material for: The Association between the Platelet to White Blood Cell Ratio and Chronic Kidney Disease in an Aging Population: A Four-Year Follow-Up Study
Source: J Clin Med. 2023 Nov 13;12(22):7073. doi: 10.3390/jcm12227073 (PMC10672662; doi:10.3390/jcm12227073)
Supplement: Supplementary file 1 [file jcm-12-07073-s001.zip › jcm-2680946-supplementary.pdf]

Supplementary file

Table S1: The cross-sectional association between PWR and prevalent CKD after interpolation

Models	PWR (continous) OR (95% CI)	PWR (as tertiles) T1 (reference)	T2 group OR (95% CI)	T3 group OR (95% CI)	*P* for trend
Model 1	0.976 (0.971-0.982) ***	1.00	0.70 (0.59-0.82) ***	0.49 (0.41-0.59) ***	<0.001
Model 2	0.979 (0.973-0.986) ***	1.00	0.74 (0.61-0.89) **	0.55 (0.44-0.67) ***	<0.001
Model 3	0.978 (0.973-0.986) ***	1.00	0.74 (0.62-0.90) **	0.55 (0.45-0.67) ***	<0.001
Model 4	0.982 (0.975-0.988) ***	1.00	0.75 (0.62-0.92) **	0.58 (0.46-0.71) ***	<0.001
Model 5	0.982 (0.976-0.989) ***	1.00	0.77 (0.63-0.94) **	0.59 (0.47-0.73) ***	<0.001

** $P < 0.01$; *** $P < 0.001$. Model 1 – crude model; Model 2 - adjusting for demographic characteristics including age, gender, marital status, educational levels and BMI; Model 3 – further adjusting for lifestyle factors including cigarette and alcohol consumption, sleep duration and afternoon nap; Model 4 – adjusting for medical histories including depression, hypertension and hyperuricemia; Model 5 – adjusting for blood biomarkers including low-density lipoprotein, high-density lipoprotein, total cholesterol, triglycerides, blood glucose and high-sensitive C-reactive protein.

Table S2: The cross-sectional association between PWR and prevalent CKD (as binary or quartiles)

Models	B1/Q1 group	Binary	Q2 group	Q3 group	Q4 group	*P* for
	Reference	OR (95% CI)	OR (95% CI)	OR (95% CI)	OR (95% CI)	trend
Model 1	1.00	0.61 (0.52-0.70) ***	0.66 (0.55-0.80) ***	0.57 (0.47-0.69) ***	0.43 (0.35-0.54) ***	<0.001
Model 2	1.00	0.66 (0.55-0.79) ***	0.70 (0.55-0.88) **	0.60 (0.47-0.77) ***	0.51 (0.39-0.66) ***	<0.001
Model 3	1.00	0.66 (0.55-0.79) ***	0.69 (0.55-0.87) **	0.60 (0.47-0.77) ***	0.50 (0.39-0.65) ***	<0.001
Model 4	1.00	0.68 (0.56-0.83) ***	0.63 (0.49-0.81) ***	0.56 (0.43-0.73) ***	0.53 (0.40-0.71) ***	<0.001
Model 5	1.00	0.69 (0.57-0.84) ***	0.63 (0.49-0.81) ***	0.57 (0.44-0.74) ***	0.54 (0.40-0.71) ***	<0.001

** $P < 0.01$; *** $P < 0.001$. Model 1 – crude model; Model 2 - adjusting for demographic characteristics including age, gender, marital status, educational levels and BMI; Model 3 – further adjusting for lifestyle factors including cigarette and alcohol consumption, sleep duration and afternoon nap; Model 4 – adjusting for medical histories including depression, hypertension and hyperuricemia; Model 5 – adjusting for blood biomarkers including low-density lipoprotein, high-density lipoprotein, total cholesterol, triglycerides, blood glucose and high-sensitive C-reactive protein.

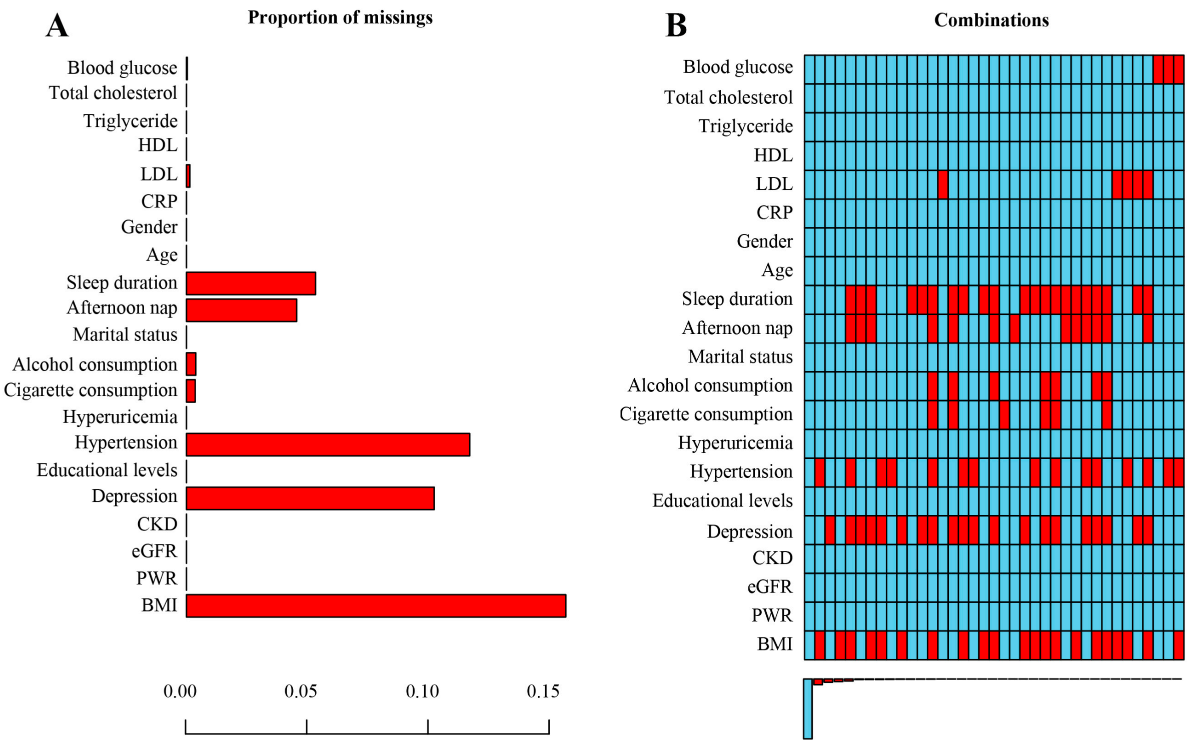

Figure S1: The missing values of covariates.

(A) The percentages of missing values of covariates; (B) The combinations of missing values of covariates.
